# HIV-1 integrase resistance among antiretroviral treatment naive and experienced patients from Northwestern Poland

**DOI:** 10.1186/1471-2334-12-368

**Published:** 2012-12-21

**Authors:** Miłosz Parczewski, Dorota Bander, Anna Urbańska, Anna Boroń-Kaczmarska

**Affiliations:** 1 Department of Infectious Diseases and Hepatology, Pomeranian Medical University, Szczecin, Poland

**Keywords:** HIV-1, Integrase inhibitors, Raltegravir, Antiretroviral treatment failure, Drug resistance mutations

## Abstract

**Background:**

HIV integrase inhibitor use is limited by low genetic barrier to resistance and possible cross-resistance among representatives of this class of antiretrovirals. The aim of this study was to analyse integrase sequence variability among antiretroviral treatment naive and experienced patients with no prior integrase inhibitor (InI) exposure and investigate development of the InI drug resistance mutations following the virologic failure of the raltegravir containing regimen.

**Methods:**

Sequencing of HIV-1 integrase region from plasma samples of 80 integrase treatment naive patients and serial samples from 12 patients with observed virologic failure on raltegravir containing treatment whenever plasma vireamia exceeded >50 copies/ml was performed. Drug resistance mutations were called with Stanford DB database and grouped into major and minor variants. For subtyping bootstrapped phylogenetic analysis was used; Bayesian Monte Carlo Marcov Chain (MCMC) model was implemented to infer on the phylogenetic relationships between the serial sequences from patients failing on raltegravir.

**Results:**

Majority of the integrase region sequences were classified as subtype B; the remaining ones being subtype D, C, G, as well as CRF01_AE , CRF02_AG and CRF13_cpx recombinants. No major integrase drug resistance mutations have been observed in InI-treatment naive patients. In 30 (38.5%) cases polymorphic variation with predominance of the E157Q mutation was observed. This mutation was more common among subtype B (26 cases, 54.2%) than non-B sequences (5 cases, 16.7%), p=0.00099, OR: 5.91 (95% CI:1.77-22.63)]. Other variants included L68V, L74IL, T97A, E138D, V151I, R263K. Among 12 (26.1%) raltegravir treated patients treatment failure was observed; major InI drug resistance mutations (G140S, Q148H and N155H, V151I, E92EQ, V151I, G163R) were noted in four of these cases (8.3% of the total InI-treated patients). Time to the development of drug resistance ranged from 2.6 to 16.3 months with mean increase of HIV viral load of 4.34 (95% CI:1.86-6.84) log HIV-RNA copies/ml at the time of emergence of the major mutations. Baseline polymorphisms, including E157Q were not associated with the virologic failure on raltegravir.

**Conclusions:**

In InI treatment naive patients polymorphic integrase sequence variation was common, with no major resistance mutants. In the treatment failing patients selection of drug resistance occurred rapidly and followed the typical drug resistance pathways. Preexisting integrase polymorphisms were not associated with the treatment failure.

## Background

HIV integrase, being one of the key retroviral enzymes necessary for successful replication, is one of attractive targets in the treatment of HIV infection. Integrase inhibitors, targeting one of the essential steps of the virus life cycle, namely strand transfer have been approved for the clinical practice in 2008, and have proven to be highly efficient in treatment of both antiretroviral-naive and experienced individuals [1-6]. As potent agents, this class of drugs is not only important part of the salvage regimens but is also useful in patients with therapy complications such as lipodystrophy, dyslipidaemia, or liver injury [7-9]. Raltegravir (RAL) is currently licensed in Europe for both treatment naive and experienced patients, elvitregravir pending EMEA approval, while newer compounds such as dolutegravir undergo phase III clinical trials [10-13].

Despite high efficacy observed among treatment experienced patients with drug resistance to other antiretroviral drug classes, low genetic barrier to resistance and possible cross-resistance among integrase inhibitors is a limiting factor in the practical use of these compounds. Virologic failure has been associated with major, signature mutations within the catalytic domain of the enzyme, and include Y143R/C, N155H Q148K/R/H integrase sequence variants associated with significant susceptibility reduction both to RAL and elvitegravir (EVG) [14-19]. Accumulation of secondary, accessory mutants is responsible for the further increase in the level of the resistance and often restored replication capacity [20-22]. Major mutations remain uncommon in the antiretroviral treatment naive patients; so far only a few cases of transmitted drug resistance (Q148H, G140S and N155H mutations) have been described [23,24]. On the other hand, polymorphic mutations in the central core domain positions have been observed in up to 34% of the published sequences [25] and 56% of the patients with recently acquired infection [26]; some of these naturally occurring variants have been observed in patients failing raltegravir and elvitegravir (L74M, T97A, S119G/R, E157Q, G163K/R), with notable the frequency variation across the subtypes [4,25,27-31]. Recent reports also describe high frequency of the minority clades bearing major and accessory mutations, however, clinical significance of this pre-existing low level variability is yet to be determined [20,27,32]. Analyses for the secondary drug resistance are currently included in the guidelines for the drug resistance testing among individuals failing integrase containing treatment [33]. This study was designed to investigate the sequence variability in the integrase region with two objectives: firstly, to characterize primary integrase resistance mutations among the treatment naive and experienced patients with no prior integrase inhibitor (InI) exposure; secondly to investigate the development of the InI drug resistance mutations following the virologic failure of the raltegravir-containing regimen.

## Methods

### Group characteristics

In this study HIV-1 integrase sequences from patients observed at the Department of Infectious Diseases and Hepatology Pomeranian Medical University, Szczecin, Poland and Out-Patient’s Clinic of Acquired Immunodeficiency, Regional Hospital, Szczecin Poland were obtained. Bioethical committee approval (Bioethical Committee of the Pomeranian Medical University, Szczecin, Poland, approval number KB-0012/08/12) was obtained for this analysis. Informed consent was provided and obtained from study participants. Eighty samples from patients who have never received the integrase inhibitors were selected, including forty-six pretreatment ones from individuals who have later received raltegravir (RAL). Additionally, sequences were obtained at the time of virologic failure on RAL (HIV-RNA levels analysed every four months), whenever such a failure have occurred. Virologic failure was defined as two consecutive viral loads >50 HIV RNA copies/ml. To assess adherence number of dispensed monthly doses of antiretroviral medication divided by the number of follow-up months, expressed as a percentage, was used.

### Sequencing

HIV RNA extraction was performed from plasma samples stored at -80 degrees Centigrade using a reagents provided with the Viroseq 2.8 kit (Abbott molecular, Abbott Park, IL, USA). HIV-1 integrase region (866 base pair, HXB2 genome location: positions 4230-5096) was amplified and sequenced with reagents and conditions specified by Laethem et al., and the following amplification and sequencing primers: AGGAGCAGAAACTTWCTATGTAGATGG (outer forward), TTCTTCCTGCCATAGGARATGCCTAAG (outer reverse), TTCRGGATYAGAAGTAAAYATAGTAACAG (inner forward), TCCTGTATGCARACCCCAATATG (inner reverse and sequencing), GCACAYAAAGGRATTGGAGGAAATGAAC (sequencing, forward), GGVATTCCCTACAATCCCCAAAG (sequencing, forward), GAATACTGCCATTTGTACTGCTG (sequencing, reverse) [34]. Amplicons obtained by the nested PCR method were used for sequencing by standard techniques with BigDye technology on an ABI 3500 platform (Applied Biosystems, Foster City, CA). Sequence assembly was performed with the Recall online tool ( http://pssm.cfenet.ubc.ca) [35]. For all the InI treated patients both baseline (prior to the raltegravir treatment) and on treatment (at the virologic failure) sequences were obtained. As integrase region sequencing has been initiated in 2011 majority of the samples have been analyzed retrospectively. Integrase sequencing in patients with viral loads >50 copies/ml was attempted from every available sample, usually collected every four months. All sequences were submitted to GenBank (accession numbers JQ305769-91, KC409134-KC409222).

### Drug resistance, subtyping and phylogenetic analyses

Drug resistance mutations were called with Stanford DB database (hivdb.stanford.edu) and grouped into major and minor mutations as assigned by this on-line tool [36]. For subtyping, phylogenetic analysis with reference sequences listed in the HIV Sequence Compendium 2011 (Los Alamos National Laboratory Los Alamos, USA http://www.hiv.lanl.gov) was used. The sequence dataset was aligned with Clustal X2.0.10 ( http://www.clustal.org). GTR+I+γ substitution model was selected with jmodeltest (software version 0.1.1) based on the lowest akaike information criterion (AIC) [37,38]. Base frequencies for the dataset were as follows: A = 0.4036, C = 0.1613, G = 0.2272, T = 0.2079, p inv parameter: 0.507, gamma shape: 0.76.

Bootstrapped (1000 replicates) maximum composite likelihood (ML) test under the GTR+I+γ model with three separate codon positions, and a nearest-neighbor-interchange ML method was inferred using MEGA 5.05 software. Phylogeny obtained subtype was compared with an on-line subtyping performed by the Stanford DB database. For clarity, phylogenetic trees were divided into subtype B and non subtype B groups and visualized with the tree explorer included in the MEGA software.

For analysis of the integrase resistance development and phylogenetic relationships between the serially obtained sequences from patients failing on raltegravir containing treatment Bayesian Monte Carlo Marcov Chain (MCMC) analyses were implemented. Two replicates of 100 million generations were run in BEAST v 1.5.3 [39], under the GTR+γ+Γ model with estimated base frequencies, gamma site heterogenity model, and three partitions for the codon positions. A consensus tree with posterior probabilities for branch support was obtained and annotated with TreeAnnotator v 1.5.4. Trees were visualized in Figtree v.1.2.2.

For nominal variables chi-square test with EPI6 Statcalc software was used (Department of Mathematics, University of Louisiana-Lafayette, Lafayette, LA, USA), while for continuous variables the Mann–Whitney U-test was implemented (Statistica software, Statsoft, Tulsa, OK, USA).

## Results

### Group characteristics

Majority of the analyzed patients were male, with HIV infection acquired by the heterosexual transmission, with clinical symptoms of immunodeficiency (either B or C according to the CDC classification), baseline lymphocyte CD4 count below 200 cells/μl and HIV-1 viral load exceeding 100 000 copies/ml (Table 1). Median age at the diagnosis for the entire analysed group was 35 years (IQR:27-45), and 37 years (IQR:26-43) for the RAL-treated patients. In twenty cases integrase inhibitor treatment was commenced at undetectable viral load, due to the toxicity of the previous regimen, drug resistance, liver failure or drug interactions.

**Table 1:** Characteristics of the study population

	Entire group (n=80)*	**Patients treated with raltegravir (n=46)**
Female gender, n (%)	34 (42.5)	19 (41.3)
*Transmission route*		
IDU, n (%)	19 (23.8)	13 (28.3)
Heterosexual, n (%)	43 (53.8)	22 (47.8)
MSM, n (%)	17 (21.3)	10 (21.7)
Unknown, n (%)	1 (1.3)	1 (2.2)
*CDC category at HIV diagnosis*		
A , n (%)	23 (28.8)	14 (30.4)
B, n (%)	34 (42.5)	19 (41.3)
C, n (%)	22 (27.5)	14 (30.4)
Lymphocyte CD4 count at diagnosis, median cells/μl, (IQR)	147 (51-331)	140 (29-336)
Lymphocyte CD4 count prior to raltegravir introduction, median cells/μl (IQR)	N.A.	335 (154-515)
HIV viral load at diagnosis, median log HIV-1 copies/ml (IQR)	5.33 ( 4.62-5.88)	5.38
		(4.62-5.56)
HIV-RNA undetectable prior to raltegravir introduction, n (%)	N.A.	20 (44)
HIV viral load prior to raltegravir introduction, median log HIV-1 copies/ml (IQR)	N.A.	4.88 (3.36-5.44)
HIV-1 subtype (integrase coding region) *		
B, n (%)	48 (61.5)	30 (68.2)
D , n (%)	24 (30.8)	10 (21.7)
C, n (%)	2 (2.6)	2 (4.5)
G, n (%)	1 (1.3)	0
CRF01_AE , n (%)	1 (1.3)	1 (2.3)
CRF02_AG, n (%)	1 (1.3)	1 (2.3)
CRF13_cpx , n (%)	1 (1.3)	0
Baseline integrase polymorphisms *		
None , n (%)	48 (61.5)	24 (54.5)
E157Q , n (%)	23 (28.8)	15 (34.1)
V151I, n (%)	2 (3.1)	1 (2.3)
E138D, n (%)	2 (2.6)	0
T97A, n (%)	1 (1.3)	1 (2.3)
R263K, n (%)	1 (1.3)	1 (2.3)
L74IL, n (%)	1 (1.3)	1 (2.3)
L68V, n (%)	1 (1.3)	1 (2.3)

*in two cases of raltegravir treated patients the integrase coding region sequencing failed, thus the subtype and interpretation of the baseline integrase polymorphisms was unavailable.

Table legend: IDU: injection drug use, MSM – men who have sex with men, IQR – interquartile range.

Integrase sequencing was consistently successful at HIV viral loads exceeding 300 copies/ml, and one sample of 134 HIV-RNA copies/ml. In two cases the sequencing failed and subtype was unavailable; as the result final dataset for the baseline integrase resistance included the group of 78 individuals.

### Subtypes and baseline drug resistance mutations

**Figure 1:**
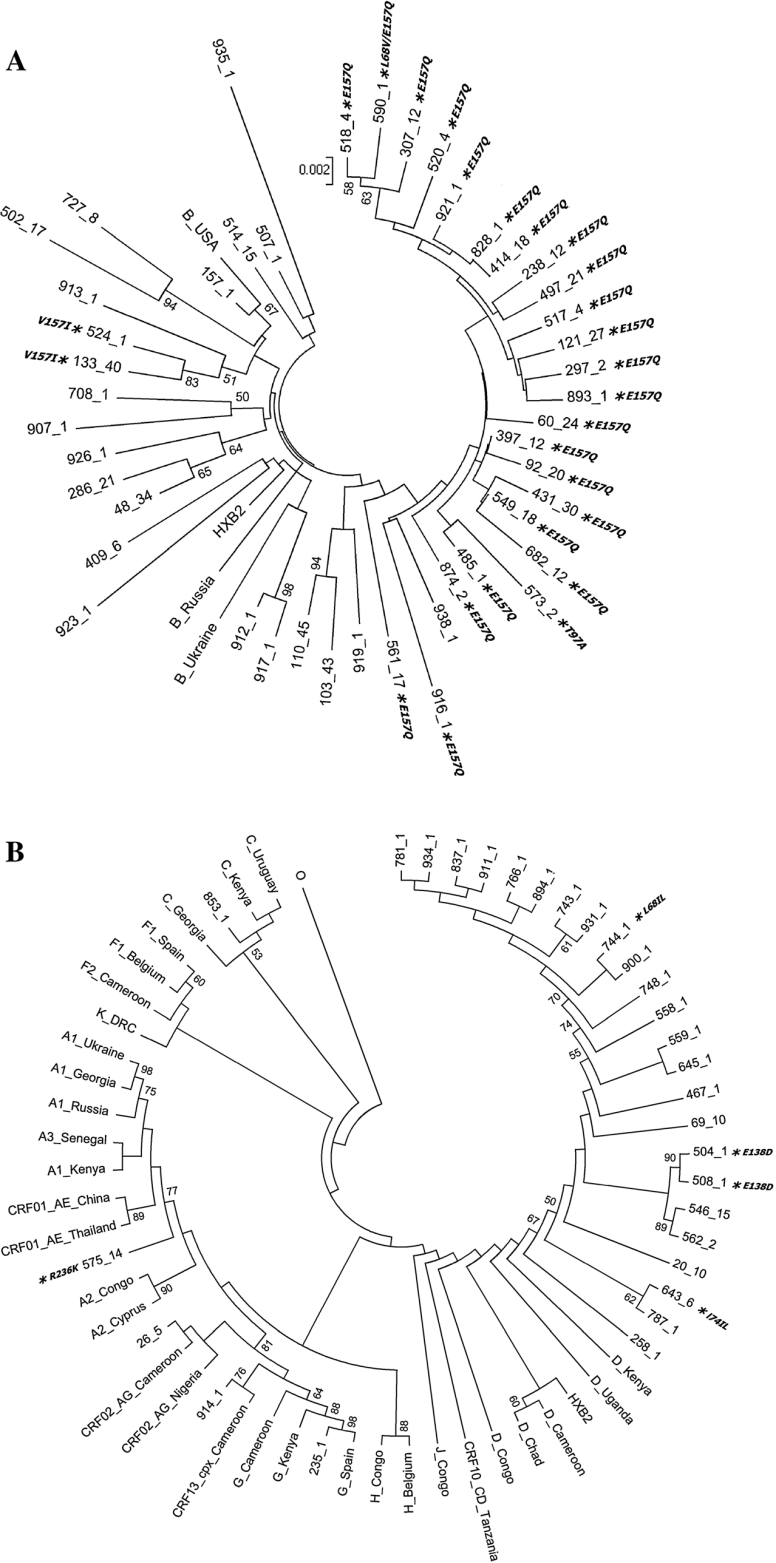
**Maximum composite likelihood inferred trees showing phylogenetic relationships of subtype B (A) and non-subtype B isolates to the HIV-1 M group reference strains.** Bootstrap values, expressed as percentage, are listed at the branch nodes. Baseline integrase mutations are listed alongside patient number following an asterisk.

Majority of the integrase region sequences were classified as subtype B; the remaining ones being subtype D, C, G, and recombinants: CRF01_AE, CRF02_AG and CRF13_cpx (Table 1, Figure 1A,B). For all but one sequence concordance between the phylogenetic analysis and Stanford DB assigned subtype was noted but CRF13_cpx recombinant was called subtype J by this on-line tool. In the subtype B infected group injection drug use associated infections were the predominant transmission route (19 cases, 39.6%), followed by the men who have sex with men (15 cases, 31.2%) and heterosexual transmissions (13 cases, 27.1%). For one subtype B infected patient the transmission route remains unknown. For all non-B subtypes heterosexual exposure was noted.

In the InI naive group no major integrase drug resistance mutations have been observed, but in 30 (38.5%) cases polymorphic variation with minimal influence on integrase inhibitor susceptibility was found, being significantly more common among subtype B sequences (26 cases, 54.2%) than non-B ones (5 cases, 16.7%), p=0.00099, OR: 5.91 (95% CI:1.77-22.63) (Figure 1a, 1b). The predominating E157Q polymorphism was found in 47.9% of the subtype B sequences and associated with injection drug use (69.6% of the sequences with E157Q were derived from the IDU patients). This variant was absent in non-B clades. Other mutations and polymorphisms observed only in subtype B integrase sequences were L68V, T97A and V151I, while in non-B sequences R263K, E138D, L68IL and L74IL variants were noted (Figure 1b, Table 1).

### Development of InI drug resistance

**Figure 2:**
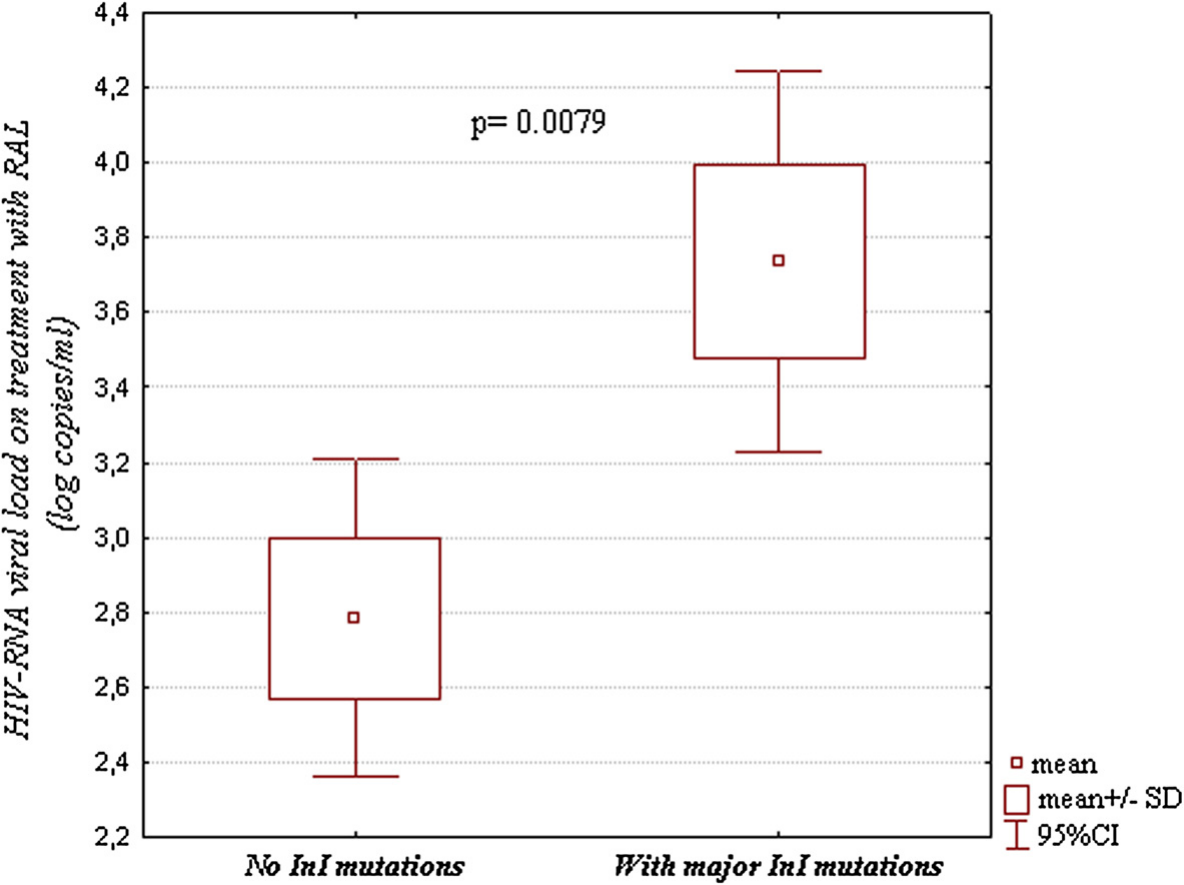
HIV-1 viral loads in the group failing raltegravir containing treatment with and without observed InI drug resistance mutations.

Of the RAL treated patients in twelve cases (26.1%) treatment failure was observed, with borderline statistical association with non-B subtype [p=0.098, OR: 3.13 (95% CI:0.64-15.7)]. In the failing group estimated adherence ranged from the 58% to 99%. In four cases (8.3% of the total number of patients treated with raltegravir, 33.3% of the failing ones) major InI drug resistance mutations have been observed. Mean viral load on raltegravir treatment in the failing group was 2.79 (95% CI:2.35-3.22) log HIV-RNA copies/ml being notably higher among patients with developed drug resistance [mean of 3.73 (95% CI:3.19-4.28) log HIV-RNA copies/ml] compared to the failing ones without the InI drug resistance mutations [mean: 2.79 (95% CI:2.35 -3.22) log HIV-RNA copies/ml] (p=0.0079, Figure 2).

Baseline polymorphisms, including the most prevalent E157Q analyzed separately, were not associated with the virologic failure on RAL [p=0.5, OR 1.56 (95% CI: 0.35-7.11) and p=0.38, OR 1.83 (95% CI:0.38-10.05), respectively]. In 14 cases with virologic success accessory mutations were present prior to the raltegravir introduction (11 sequences with E157Q, one of each L68V/E157Q, T97A, V151I). Among virologically failing patients E157Q was noted in one patient with N155H mutant, in three E157Q variant was present at baseline and consistently in the sequences obtained on RAL therapy while in two patients either R263K or L74IL were present at baseline and disappeared in the subsequent sequences on virologically unsuccessful treatment.

**Figure 3:**
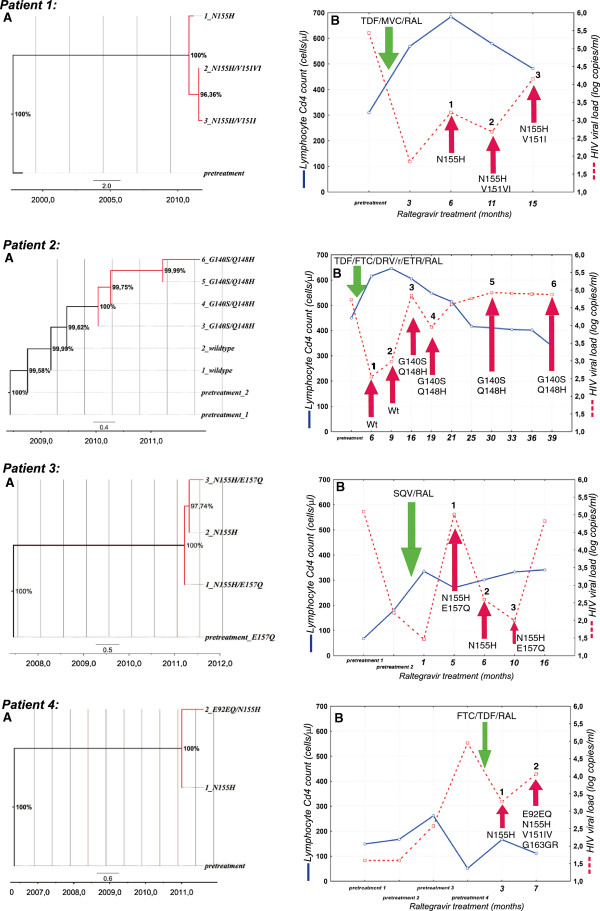
**(A) Phylogenetic trees (time-annotated MCMC) of the serial sequences and (B) HIV-1 viral loads and lymphocyte CD4 counts from four patients failing raltegravir (RAL) treatment.** Maximum likelihood tree with bootstrap values for 1000 replicates drawn at the tree branches. Integrase resistance mutations are marked at the tip nodes; branches with developed drug resistance are marked red. Time points in which InI drug resistance mutations were noted are indicated with red arrows, while initiation of the raltegravir containing antiretroviral treatment is indicated with green arrow. TDF – tenofovir, MVC- maraviroc, FTC – emticitabine, DRV/r – ritonavir boosted darunavir, SQV – saquinavir.

Development of drug resistance mutations followed two patterns: major N155H with or without subsequent accessory V151I, E92EQ, V151I, G163R mutants (three cases) and Q148H accompanied by G140S mutant (one case) (Figure 3B). To determine the intra-patient relationships between the mutations we have also used a time-annotated phylogenetic Bayesian Monte Carlo Marcov Chain analysis rooted with the pretreatment samples (Figure 3A). Time to the development of drug resistance ranged from the 2.6 to 16.3 months with mean increase of the HIV viral load (available for patients 1-3) of 4.34 (95% CI:1.86-6.84) log HIV-RNA copies/ml at the time point of emergence of the major drug resistance mutations.

It must be noted, that three of the patients with developed drug resistance were heavily experienced with reverse transcriptase (RT) and protease (PR) mutations (patient 1: RT: M41L, K103N, M184V, T215S; PR: L10I; patient 2: PR: M41L, V118I, K103N, M184V, L210W, T215S, RT: L10I, M46I, I54V, L63P, A71T, V82A, L90M, patient 4: RT: K103N, M184V, P225H, F227L), while one patient (number 3) due to endocarditis and kidney failure was treated with live-saving but suboptimal therapy which consisted solely of ritonavir-boosted saquinavir and raltegravir.

## Discussion

Integrase inhibitors remain an attractive option and have become a vital component of the modern antiretroviral treatment, especially among patients with preexisting drug resistance or treatment complications [2,40-45]. It is necessary to monitor the transmission and *de novo* development of drug resistance mutations decreasing the susceptibility of HIV against this class of antiretrovirals to provide the virologists and clinicians with the current data allowing for adequate therapeutic strategies.

In the presented study, among the InI naive patients no major drug resistance mutations have been observed, however, accessory mutations have been common (38.5%). Of the noted variants, four (L68V, T97A, V151I, E157Q) have previously been described as polymorphic, occurring in >1% of integrase sequences [25]. These integrase mutations were more prevalent in the subtype B viruses with E157Q occurring at higher frequencies than previously reported [46-50]. In the previous reports this polymorphism was shown to impair the integrase 3′end processing and strand transfer [17] but was associated with only minimal reduction of the susceptibility to RAL and elvitegravir (<6 fold) [25,26,51,52]. Of note, in our study the E157Q mutation was observed mostly among phylogenetically related subtype B infected intravenous drug users (Figure 1A), and was not associated with higher ratio of the virological failure. This is the largest described so far cluster with this polymorphism in subtype B infected patients. Lack of clinically important, primary resistance mutations for raltegravir, elvitegravir and dolutegravir is consistent with published reports from other studies [27,47,53-59], and is supports the fact that the transmission of the drug resistance is unlikely in the populations previously unexposed to the integrase inhibitor treatment [60].

In the group of the RAL treated patients treatment was successful in 73.9% of cases, while the 8.3% of the failing patients developed major drug resistance mutations significantly reducing susceptibility to both raltegravir and elvitegravir. Number of virologic failures was higher than observed in the STARTMRK and BENCHMRK trials for the patients with high baseline genotypic scores [1,5,6,15], however, in the analysed group integrase inhibitor was usually considered as a second line treatment and was selected due to toxicity, drug resistance or drug-drug interactions, as well as preexisting drug resistance in treatment experienced patients; suboptimal adherence was also commonly noted - only in three (one described below with developed integrase resistance mutations and two with integrase-inhibitor susceptible variants) failing patients estimated adherence exceeded 90%. Another reason for the high frequency of treatment failure on raltegravir-containing regimen may be related to the preexisting drug resistance mutations in the reverse transcriptase region resulting in the lower susceptibility to the background regimen. NRTI drug resistance mutations (thymidyne analog mutations and M184V) were present in six (50%) of the twelve treatment failing patients prior to RAL introduction. Additionally, treatment efficacy was numerically worse among non-B subtypes (with borderline statistical significance), however this may be associated with small group size as in the previous reports raltegravir was comparably efficacious across B and non-B HIV-1 subtypes [4].

Integrase drug resistance profiles in four treatment failing patients with drug resistance followed two typical pathways, Q148H/G140S (one case) and N155H/V151I (three patients), associated with high level resistance to raltegravir and cross-resistance to elvitegravir [16-18,61,62]. Similar cases have been described previously [19,61,63]. Of note, in the patient with the Q148H/G140S salvage treatment option with dolutegravir was also lost due to the resistance associated with this pathway [64]. As expected, in cases observed in our study virologic rebound was associated with significant increase of the plasma viral load at the time-point of the occurrence of the major mutation. Stepwise accumulation of mutations was observed in two patients with N155H followed by the secondary E92Q, V151I and/or G163R mutations resulting in further increase raltegravir and elvitegravir resistance and often enhanced replicative capacity [27,65]. All observed mutations have been previously associated with raltegravir drug resistance observed in *in vivo* studies [15,45]. The N155H pathway is also associated with smaller reduction in the RAL susceptibility than the Q148H, moreover, double Q148H/G140S mutants were found to be fitter than the E92Q/N155H ones [66,67]. This is consistent with the observed higher viral loads at the time of the development of drug resistance in a patient 2 (if compared to the patients 1,3,4) in whom such a highly fit double mutant was observed (Figure 3). In the patient 3 the observed drug resistance is probably associated with the suboptimal treatment and poor drug exposure when combined only with boosted saqinavir, however no other treatment option was possible for the patient at the time of therapy initiation . Time to InI resistance development in the observed patients did not exceed few months, except for one case with the double Q148H/G140S mutant, which is in accordance to the data on the low genetic barrier for the integrase inhibitors and rapid selection of the resistant variants [15,68].

## Conclusions

In summary, the results of this study provide data for the clinical practice and the treatment with integrase inhibitors. No major mutations associated with integrase dug resistance have been found in the pretreatment samples, however the polymorphic variation was common. In the failing patients selection of drug resistance occurred rapidly, and followed the typical pathways with accumulation of the drug resistance. Poor adherence, preexisting drug resistance and suboptimal combination were the key reasons associated with the development of the drug resistance.

## Competing interests

The authors declare that they have no competing interests related to this study.

## Authors’ contributions

Conceived and designed the study: MP. Performed the experiments and analyses: MP AU. Analyzed the data: MP. Contributed reagents/materials/analysis tools: MP AU. Statistical and phylogenetic analyses: MP. Wrote the paper: MP DB AU ABK. All authors read and approved the final manuscript.

## Pre-publication history

The pre-publication history for this paper can be accessed here:

http://www.biomedcentral.com/1471-2334/12/368/prepub
